# Association of serum ADAMTS-7 levels with left ventricular reverse remodeling after ST-elevation myocardial infarction

**DOI:** 10.1186/s40001-018-0305-1

**Published:** 2018-03-10

**Authors:** Wenjing Wu, Jiahui Li, Changan Yu, Yanxiang Gao, Shuying Fan, Xiaojun Ye, Yong Wang, Jingang Zheng

**Affiliations:** Department of Cardiology, China-Japan Friendship Hospital, Beijing, 100029 China

**Keywords:** Acute myocardial infarction, ADAMTS-7, Left ventricular reverse remodeling

## Abstract

**Background:**

Left ventricular reverse remodeling (LVRR) in patients with ST-elevation myocardial infarction (STEMI) is associated with a good prognosis. Serum levels of ADAMTS-7 might be used for the prognosis of STEMI. This study aimed to investigate the relationship between serum ADAMTS-7 levels and LVRR.

**Methods:**

This was a prospective study of 104 patients with STEMI who underwent revascularization and 63 controls. ADAMTS-7 serum levels were measured on days 1, 3, and 7 and in months 1 and 6 after STEMI. A decrease ≥ 15% of the left ventricular end-systolic volume at 6 months was defined as LVRR.

**Results:**

The serum levels of ADAMTS-7 in patients with LVRR were lower than those without LVRR (3.84 ± 2.26 vs. 5.02 ± 2.54, *P* = 0.032) 7 days after STEMI and the difference between day 7 and day 1 (ΔADAMTS-7) was even significantly lower (− 1.31 ± 0.94 vs. − 0.30 ± 0.22, *P* = 0.021). Multivariate analysis showed that ΔADAMTS-7_(day 7 minus day 1)_ was independently associated with LVRR (OR = − 0.322, 95% CI = − 0.996 to − 0.074, *P* = 0.028). Receiver operating characteristic (ROC) curve analysis showed that LVRR could be predicted (sensitivity 89%, specificity 82%, and area under the curve 0.896) when ΔADAMTS-7_(day 7 minus day 1)_ was < − 0.39.

**Conclusions:**

ΔADAMTS-7_(day 7 minus day 1)_ might be a potential predictive factor for LVRR.

## Background

ST-elevation myocardial infarction (STEMI) is a leading cause of heart failure and has a seriously adverse prognosis [1]. Left ventricular reverse remodeling (LVRR) is a dynamic process of myocardial repair and usually takes a few months [2]. LVRR is often accompanied with left ventricular volume decrease and left ventricular ejection fraction (LVEF) improvement [3]. It was reported that the 2-year incidence of cardiovascular endpoint events was decreased by 20% in patients with acute myocardial infarction (AMI) who achieved LVRR [3]. Therefore, LVRR is a factor of good prognosis after STEMI.

It was suggested that the degradation of the extracellular matrix (ECM) was involved in the occurrence of ventricular remodeling after AMI [4]. Matrix metalloproteinases (MMPs) degrade the ECM and were found to be involved in ventricular remodeling [5]. However, no specific MMP inhibitor is currently available and broad-spectrum MMPs inhibitors impair the normal structure of tissues [6]. Therefore, the interest in MMPs and MMP inhibitors for the treatment of patients with AMI faded in the recent years [7].

A disintegrin and metalloproteinase with thrombospondin motifs (ADAMTS) is a family of 19 peptidases recently found to degrade ECM in a similar way than MMPs, but with a narrower substrate spectrum and higher specificity [8], making these enzymes proper targets for therapy with higher safety profile than MMPs [9]. ADAMTS-7 was reported to degrade the ECM through the degradation of cartilage oligomeric matrix proteins (COMP), which is present in the skeletal muscle system and also found to be a component of human ECM [10]. ADAMTS-7 is involved in vascular reconstruction through influencing the migration of smooth muscle cells and intimal neovascularization [11]. Upregulation of ADAMTS-7 could lead to calcification of vascular smooth muscle cells [12]. A previous study by our group showed that the serum levels of ADAMTS-7 were significantly increased in patients with AMI with LVEF < 35% and were an independent predictor for heart failure after AMI [13]. However, there is no report on the relationship between serum levels of ADAMTS-7 and LVRR after STEMI.

Given the fact that serum levels of ADAMTS-7 and LVRR are associated with prognosis after STEMI, the relationship between ADAMTS-7 and LVRR is of interest. In the present study, the association between serum levels of ADAMTS-7 and LVRR after STEMI was investigated.

## Methods

### Study design

This was a single center prospective cohort study of 104 patients with ST-elevation myocardial infarction (STEMI) consecutively recruited between December 2012 and December 2015. All study procedures and data collections were performed at the China-Japan Friendship Hospital (Beijing, China). All patients successfully underwent reperfusion therapy within the therapeutic window (i.e., within 12 h after onset). The STEMI diagnosis and reperfusion therapy were in accordance with the treatment guidelines for STEMI [14]. Exclusion criteria were: (1) tumor; (2) dysfunction of liver or kidney; (3) any infection or elevated markers of inflammation at the time of blood sampling; (4) peripheral artery disease; or (5) surgery within 6 months. Thus, 63 subjects who had no history of cardiovascular diseases were recruited during routine physical examinations.

The study was approved by the ethics committee of the China-Japan Friendship Hospital. An informed consent was signed by each participant before entering the study.

### Interventions

Preoperative aspirin and clopidogrel were given to all patients. The culprit vessel was defined according to clinical manifestation, echocardiography, and angiography [15]. A drug-eluting stent was implanted. The standard postoperative medication included angiotensin-converting enzyme inhibitor (ACEI), angiotensin receptor blocker (ARB), β receptor blocker, aldosterone antagonists, statins, aspirin, and clopidogrel. All subjects received counseling about lifestyle improvements. There were no serious adverse cardiovascular events such as revascularization, fatal arrhythmias, stent thrombosis, hospitalization for any reason, or all-cause mortality within the 6-month follow-up. Stent restenosis was observed in 3% of the patients of both groups.

### Data collection

Demographic (age, sex), clinical [body mass index (BMI), hypertension, diabetes, hyperlipidemia, smoking], and intervention (culprit vessel, door-to-balloon time, restenosis) variables were collected after intervention and during follow-up.

### Biochemistry

Blood was sampled on days 1, 3, and 7, and in months 1 and 6 after STEMI. Baseline serum creatinine, hemoglobin, hematocrit, and high-sensitivity C-reactive protein (hsCRP) were routinely measured before and after PCI. Baseline fasting blood glucose, blood lipids, and HbA1c were tested in the morning of the first day after admission. Creatinine kinase (CK) peak, CK-MB peak, and troponin I (TNI) peak were measured every 4 h until the values started to decrease. Plasma B-type natriuretic peptide (BNP) levels were determined on days 1, 3, 5, and 7 after admission. All the laboratory tests were conducted at the central laboratory of cardiovascular diseases of the China-Japan Friendship Hospital.

### Plasma ADAMTS-7 and BNP measurement

The blood samples (6 mL) were obtained from the brachial artery and stored at 4 °C in EDTA anti-coagulation tubes. The plasma was obtained after centrifugation for 15 min at 1000×*g* for 30 min. Samples were stored at − 80 °C.

ADAMTS-7 serum levels were tested by enzyme-linked immunosorbent assay (ELISA) using the Human ADAMTS-7 ELISA kit (MyBioSource Inc., San Diego, CA, USA) and an ELISA reader (Spectramax M2;Molecular Devices, USA). The optical density value was obtained at 450 nm. The detection threshold was 1.529 ng/mL.

The BNP immunofluorescence assay kit was from Hengzhong Biotechnology Co., Ltd. (Shijiazhuang, China). The linear range is 5–5000 pg/mL. The plate was read using a Triage Meter Plus fluorescence immunoassay analyzer (Biosite Incorporated, San Diego, CA, USA).

### Echocardiography

Cardiac structure and function were assessed by two-dimensional transthoracic echocardiography within 24 h of STEMI and after discharge for 6 months using a Vivid E9 (GE Healthcare, Waukesha, WI, USA) and two high-resolution M5S probes to acquire two-dimensional apical four-chamber and two-chamber views. The endocardial boundary was manually depicted based on the definition of electrocardiographic *R* peak as a marker of end diastolic stage and terminal *T* wave as the end of the systolic stage.

The left ventricular end-diastolic volume (LVEDV), left ventricular end-systolic volume (LVESV), and left ventricular eject fraction (LVEF) were measured according to the Simpson’s method [16]. LVRR was defined as the decrease of LVESV at 6 months, as (LVESV_0 month_ − LVESV_6 months_)/LVESV_0 month_ ≥ 15% [3].

### TIMI score at admission

The TIMI score was determined at admission, as previously described [14, 17]. The TIMI score of patients with STEMI [14], was evaluated according to the seven risk factors, one score for each risk, including: age ≥ 65 years old; at least three risk factors of coronary heart disease; previous coronary artery stenosis ≥ 50%; electrocardiographic ST segment alteration; at least two episodes of angina pectoris within 24 h; aspirin taken 7 days before the onset of AIM; and elevated levels of myocardial markers [17].

### Statistical analysis

SPSS 17.0 (IBM, Armonk, NY, USA) was used for statistical analysis. All data were tested using the Shapiro–Wilk normality test. If normally distributed, data were expressed as mean ± standard deviation (SD) and compared using independent samples *t* test. If non-normally distributed, data were expressed as median (range) and analyzed using the Mann–Whitney *U* test. Generalized estimating equations were used to evaluate the difference in the time-dependent ADAMTS-7 changes with the *P* value adjusted according to Bonferroni’s multiple pairwise method. Categorical data were presented as frequencies and compared using the Fisher’s exact test. Odds ratio (OR) and 95% confidence interval (95% CI) were calculated using multivariate stepwise logistic regression analysis using LVRR at 6 months as the dependent variable. Correlation of serum ADAMTS-7_(day 7)_ levels and ΔADAMTS-7_(day 7 minus day 1)_ with the change in left ventricular parameters from day 1 to 6 months after STEMI was analyzed using the spearman rank correlation coefficient. The diagnostic value, sensitivity, and specificity of ΔADAMTS-7_(day 7 minus day 1)_ as a predictive factor were evaluated using receiver operating characteristic (ROC) curves. Two-tailed *P*-values < 0.05 were considered statistically significant.

## Results

### Clinical characteristics

Fifty-six patients (54%) with LVESV ≥ 15% 6 months after STEMI were grouped as LVRR, and 48 patients (46%) were grouped as non-LVRR. There was no difference in demographic or clinical features between the two groups (Table 1). All the patients were treated with dual anti-platelets, ACEI, β blockers, and statins. Controls were aged 67.4 ± 9.2 years and 65% were men.

**Table 1:** Characteristics of the patients with STEMI

Variables	LVRR (*n* = 56)	Non-LVRR (*n* = 48)	*P*
Age (years)	66.6 ± 8.7	68.2 ± 10.1	0.52
Male, *n* (%)	38 (67.9)	36 (75.0)	0.44
Body mass index (kg/m^2^)	23.2 ± 3.4	24.6 ± 3.2	0.89
Hypertension history, *n* (%)	38 (67.9)	30 (62.5)	0.48
Diabetes history, *n* (%)	32 (57.1)	27 (56.2)	0.31
History of hyperlipidemia, *n* (%)	29 (51.8)	27 (56.2)	0.26
Smoking history, *n* (%)	18 (32.1)	18 (37.5)	0.37
Culprit vessel, anterior descending branch, *n* (%)	29 (51.8)	24 (50.0)	0.53
Single vessel, *n* (%)	38 (67.9)	33 (68.8)	0.61
Door-to-balloon time (min)	85.5 ± 38.4	84.3 ± 38.0	0.91
Restenosis, *n* (%)	3 (5.4)	3 (6.3)	0.66
CK peak (IU/L)	2537 ± 1268	2664 ± 1067	0.73
CK-MB peak (IU/L)	202 ± 165	269 ± 174	0.33
TNI peak (ng/mL)	22.47 ± 6.01	24.36 ± 4.20	0.62
Serum creatinine (mol/L)	87.2 ± 18.57	93.2 ± 34.16	0.27
Hemoglobin (g/dL)	13.7 ± 1.5	13.6 ± 1.4	0.19
Hematocrit (%)	57.2 ± 14.3	46.8 ± 5.6	0.29
Fasting blood glucose (mmol/L)	8.42 ± 4.63	8.44 ± 4.96	0.50
Glycosylated hemoglobin (%)	6.47 ± 1.73	6.36 ± 1.25	0.66
Serum cholesterol (mmol/L)	4.88 ± 1.79	4.92 ± 1.21	0.58
High density lipoprotein cholesterol (mmol/L)	1.04 ± 0.22	1.07 ± 0.32	0.54
Low density lipoprotein cholesterol (mmol/L)	3.62 ± 1.22	3.57 ± 1.24	0.74
hsCRP (mg/L)	0.56 ± 0.39	0.57 ± 0.34	0.87
TIMI score	2.84 ± 1.22	2.69 ± 1.16	0.51
LVEDV (mL)	190.1 ± 29.6	181.4 ± 38.3	0.71
LVESV (mL)	92.8 ± 26.5	85.2 ± 33.1	0.34
LVEF (%)	52.3 ± 8.5	53.5 ± 12.1	0.52
Plasma BNP peak levels (pg/mL)	168.4 ± 129.8	223.7 ± 213.5	0.35

*CK* creatinine kinase, *CK-MB* MB fraction of creatinine kinase, *TNI* troponin I, *hsCRP* high sensitivity C-reactive protein, *TIMI* thrombolysis in myocardial infarction, *LVEDV* left ventricular end-diastolic volume, *LVESV* left ventricular end-systolic volume, *LVEF* left ventricular ejection fraction, *BNP* B-type natriuretic peptide

### ADAMTS-7 levels after STEMI

Table 2 presents the time course of serum ADAMTS-7 levels after STEMI. There were no significant differences in ADAMTS-7 levels before and 1 day after surgery. The serum levels of ADAMTS-7 in patients with STEMI were significantly higher than in controls (all *P* < 0.05). Serum ADAMTS-7 reached its nadir 3 days after STEMI in 42 patients out of 104 (40%), and the other 62 patients (60%) showed their nadir of ADAMTS-7 levels 7 days after STEMI. Then the serum levels of ADAMTS-7 gradually increased until 6 months after STEMI (7.24 ± 3.94 ng/mL).

**Table 2:** Time course of serum ADAMTS-7 levels in patients with STEMI

	STEMI (*n* = 104)						Controls (*n* = 63)
	Day 0	Day 1	Day 3	Day 7	1 month	6 months	
Serum ADAMTS− 7 (ng/mL)	5.03 ± 2.99	5.14 ± 3.06	4.43 ± 2.19	4.32 ± 2.42	5.02 ± 2.83	7.24 ± 3.94	1.62 ± 1.43
*P* (vs. day 1)	0.083	–	0.024	0.026	0.31	0.042	0.001
*P* (vs. controls)	0.006	0.006	0.012	0.009	0.008	0.002	–

### ADAMTS-7 level comparison between STEMI patients with and without LVRR

Serum levels of ADAMTS-7 were significantly decreased 1 week after STEMI in patients with LVRR compared with those without LVRR (3.84 ± 2.26 vs. 5.02 ± 2.54 ng/mL, *P* = 0.032) (Table 3). The ΔADAMTS-7_(day 7 minus day 1)_ in patients with LVRR was also significantly decreased (− 1.31 ± 0.94 vs. − 0.30 ± 0.22 ng/mL, *P* = 0.021) (Table 3).

**Table 3:** Comparison of serum levels of ADAMTS-7 and ΔADAMTS-7 between patients with and without LVRR

Group	Serum ADAMTS-7 levels (ng/mL)					ΔADAMTS-7 (ng/mL)^a^			
	Day 1	Day 3	Day 7	1 month	6 months	Day 3	Day 7	1 month	6 months
LVRR (*n* = 56)	5.33 ± 3.22	4.27 ± 2.32	3.84 ± 2.26	5.01 ± 2.94	7.09 ± 3.89	− 1.04 ± 0.27	− 1.31 ± 0.94	− 0.29 ± 0.84	1.79 ± 1.64
Non-LVRR (*n* = 48)	5.18 ± 3.10	4.89 ± 2.21	5.02 ± 2.54	5.04 ± 2.68	7.36 ± 3.96	− 0.59 ± 0.21	− 0.30 ± 0.22	0.04 ± 0.61	2.22 ± 1.96
*P*	0.079	0.085	0.032	0.242	0.241	0.053	0.021	0.143	0.086

*LVRR* left ventricular reverse remodeling

^a^Calculated as the tested time point minus day 1

### Correlation of serum ADAMTS-7 levels 1 week after STEMI and ΔADAMTS-7_(day 7 minus day 1)_ with changes in LV functional parameters from day 1 to 6 months after STEMI

ΔADAMTS-7_(day 7 minus day 1)_ was positively correlated with ΔLVEDV_(6 months minus day 1)_ (*r* = 0.646, *P* = 0.029), ΔLVESV_(day 7 minus day 1)_ (*r* = 0.693, *P* = 0.017), ΔLVEF_(6 months minus day 1)_ (*r* = − 0.523, *P* = 0.018), and ΔBNP_(6 months minus day 1)_ (*r* = 0.701, *P* = 0.031) (Table 4).

**Table 4:** Correlation of serum ADAMTS-7_(day 7)_ levels and ΔADAMTS-7_(day 7 minus day 1)_ with the change in left ventricular parameters and BNP from day 1 to 6 months after STEMI

Variable	Serum ADAMTS-7_(day 7)_ (ng/mL)		ΔADAMTS-7_(day 7 minus day 1)_ (ng/mL)	
	*r*	*P*	*r*	*P*
ΔLVEDV (mL)	0.573	0.043	0.646	0.029
ΔLVESV (mL)	0.467	0.075	0.693	0.017
ΔLVEF (%)	− 0.265	0.262	− 0.523	0.018
ΔBNP (pg/mL)	0.648	0.039	0.701	0.031

*LVEDV* left ventricular end-diastolic volume, *LVESV* left ventricular end-systolic volume, *LVEF* left ventricular ejection fraction, *BNP* B-type natriuretic peptide

### Association of ΔADAMTS-7_(day 7 minus day 1)_ with LVRR 6 months after STEMI

**Fig. 1:**
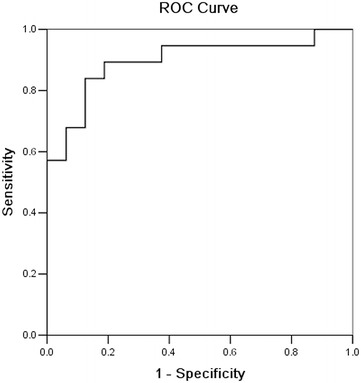
Receiver operating characteristic (ROC) curve for the prediction of left ventricular reverse remodeling in patients with ST-elevation myocardial infarction. ΔADAMTS-7_(day 7 minus day 1)_ (cut-off value: − 0.39 ng/mL; AUC: 0.896; sensitivity: 89%; specificity: 82%)

Multivariate logistic regression analysis showed that ΔADAMTS-7 was independently associated with LVRR in patients with STEMI (OR = − 0.322, 95% CI = − 0.996 to − 0.074, *P* = 0.028), while CK-MB peak and ΔBNP _(6 months minus day 1)_ were not (OR = − 0.095, 95% CI = − 0.375 to 0.221, *P* = 0.282 and OR = − 0.129, 95% CI = − 0.383 to 0.156, *P* = 0.137, respectively). The ROC curve analysis for the prediction of LVRR in patients with STEMI showed that ΔADAMTS-7_(day 7 minus day 1)_ at a cut-off value of − 0.39 ng/mL had an area under the curve of 0.896, sensitivity of 89%, and specificity of 82% (Fig. 1).

## Discussion

LVRR in patients with STEMI is closely associated with prognosis [3]. Compared with MMPs, ADAMTS-7 has a narrower substrate spectrum and might be a better pharmacological target [10]. In addition, serum levels of ADAMTS-7 might be used for the prognosis of AMI [13], but the contribution of ADAMTS-7 to LVRR has not been reported. Therefore, the present study aimed to investigate the relationship between serum ADAMTS-7 level and LVRR after STEMI. Results showed that serum ADAMTS-7 levels in patients with STEMI were significantly higher than in controls. They gradually decreased from day 3 to day 7 and then gradually increased at 6 months after STEMI. Serum ADAMTS-7 levels 1 week after STEMI and ΔADAMTS-7_(day 7 minus day 1)_ were significantly decreased in patients with LVRR compared with those without LVRR. ΔADAMTS-7_(day 7 minus day 1)_ was positively correlated with ΔLVEDV_(6 months minus day 1)_ and ΔLVESV_(6 months minus day 1)_, and negatively correlated with ΔLVEF_(6 months minus day 1)_. ΔADAMTS-7_(day 7 minus day 1)_ was an independent risk factor for LVRR after STEMI.

The ADAMTS family belongs to the inflammatory factors and plays critical roles in many diseases [18]. In this study, ADAMTS-7 levels were significantly increased at the early stage of AMI, probably because of plaque rupture and inflammatory factors being released [19]; ADAMTS-7 levels gradually decreased from day 3 to day 7 and gradually increased afterwards. ADAMTS-7 is considered to participate in the degradation of ECM mediated by COMP, which facilitates LVRR [13]. There were no significant differences of serum ADAMTS-7 between patients with and without LVRR on the first day after STEMI, but ADAMTS-7 levels were significantly decreased in patients with LVRR 1 week later, suggesting that ADAMTS-7 decrease is involved in LVRR, which is supported by a previous study [13] and by results in other MMPs [20]. The analysis showed that ΔADAMTS-7 was not influenced by the clinical characteristics, as previously shown [13], and that LVRR was the only factor affected by the serum levels of ADAMTS-7. Therefore, it might be speculated that ΔADAMTS-7_(day 7 minus day 1)_ might reflect LVRR as an early diagnostic factor. Indeed, this present study suggests that patients with ΔADAMTS-7 ≥ − 0.39 might be used to guide the management of these patients. Nevertheless, because the involvement of ADAMTS-7 in LVRR has only been recently described, additional studies are necessary to determine its exact role and contribution in LVRR. In addition, since other MMPs are also associated with LVRR, more comprehensive studies should examine these factors together. Indeed, a recent study showed that ΔMMP-2_(day 7 minus day 1)_ was also associated with LVRR and that a ΔMMP-2_(day 7 minus day 1)_ of < − 158.5 ng/mL predicted LVRR with 91.7% sensitivity and 76.9% specificity [20]. Combining different MMPs could yield an even better accuracy.

The exact mechanisms of ADAMTS-7 for cardiac remodeling are poorly known. ADAMTS-7 is widely expressed in the heart and large vessels, but knockout of ADAMTS-7 under physiological conditions has no significant effect on the cardiac structure and function of mice [21]. There is little expression of ADAMTS-7 in the myocardium rats 28 days after myocardial infarction [22]. Cultured synovial fibroblasts from healthy individuals and patients with osteoarthritis can secrete ADAMTS-7 [23]. Cartilage oligomeric matrix proteins (COMP) can induce the expression of type I collagen in hepatic stellate cells [24]. C-end spherical areas of COMP can be connected to types I, II, and IX collagen, suggesting that they may be involved in maintaining the integrity of the collagen network. Another important function of COMP is to assist the secretion of type I collagen [25]. Collagen content is low in the ECM after AMI [25] and the ventricular cavity is enlarged and the ventricular wall is thinned [26]. Therefore, it was suspected that the decrease in COMP levels after myocardial infarction promoted ventricular dilatation. The degradation of COMP in cardiac fibroblasts could result in a decrease in type I collagen secretion that changes ECM components and promotes ventricular dilatation [25]. ADAMTS-7 includes four TSP motifs domains at C-end, which are binding sites for COMP [27]. ADAMTS-7 cannot degrade COMP in ECM in various tissues [12, 28], but can degrade COMP in synovial fibroblasts [23]. Thus, ADAMTS-7 may change ECM components and promote ventricular dilatation by promoting COMP degradation in cardiac fibroblasts. Nevertheless, the exact mechanisms still require confirmation, but the present showed that the ADAMTS-7 levels correlated with BNP levels, a well-known marker of myocardial damage. This association is supported by previous studies [13, 29, 30].

This study is not without limitations. Firstly, the sample size was small and from a single center, and the follow-up period was relatively short. Patients with symptoms of STEMI but normal angiography could be included in the future as supplementary controls. Secondly, ADAMTS-7 was only tested in the peripheral blood and not in the myocardial tissues. Thirdly, the mechanisms of ΔADAMTS-7_(day 7 minus day 1)_ remain to be elucidated, as well as the effect of circulating inflammatory factors on ADAMTS-7 expression. Fourthly, it is also unknown whether some drugs such as ACEI or ARBs influence the levels of ADAMTS-7, but this factor is unlikely to have affected the results of the present study since all patients were treated with the same drugs and according to the same guidelines. Fifthly, because rheumatism is not routinely examined at our department, patients with rheumatisms were not excluded even if rheumatism could have affected the results [28], but patients with any infection at the time of blood sampling were excluded, and the prevalence of rheumatism in the Chinese population is not high [31]. Nevertheless, the cut-off value of ΔADAMTS-7_(day 7 minus day 1)_ was − 0.39 ng/mL, for a positive rate in the LVRR group of 94.6%. In addition, echocardiography revealed no overt case of rheumatic heart disease. Studies with a larger sample size and the corresponding fundamental studies on the mechanisms of ΔADAMTS-7_(day 7 minus day 1)_ are needed to confirm our findings.

## Conclusions

In conclusion, ΔADAMTS-7_(day 7 minus day 1)_ was independently associated with LVRR after STEMI, and might be a potential predictive factor for LVRR.

## Abbreviations

LVRR: left ventricular reverse remodeling
AMI: acute myocardial infarction
LVESV: left ventricular end-systolic volume
ECM: extracellular matrix
ADAMTS: a disintegrin and metalloproteinase with thrombospondin motifs
COMP: cartilage oligomeric matrix proteins
ACEI: angiotensin-converting enzyme inhibitor
ARB: angiotensin receptor blocker
BMI: body mass index
ELISA: enzyme-linked immunosorbent assay
BNP: B-type natriuretic peptide
LVEDV: left ventricular end-diastolic volume
LVEF: left ventricular eject fraction
NSTEMI: non-ST-elevation myocardial infarction
UAP: unstable angina
SD: standard deviation
STEMI: ST-elevation myocardial infarction
ROC: receiver operating characteristic
